# Triptolide Preserves Cognitive Function and Reduces Neuropathology in a Mouse Model of Alzheimer's Disease

**DOI:** 10.1371/journal.pone.0108845

**Published:** 2014-10-02

**Authors:** Shaowu Cheng, Kyle J. LeBlanc, Ling Li

**Affiliations:** Department of Experimental and Clinical Pharmacology, University of Minnesota, Minneapolis, Minnesota, United States of America; University of S. Florida College of Medicine, United States of America

## Abstract

Triptolide, a major bioactive ingredient of a widely used herbal medicine, has been shown to possess multiple pharmacological functions, including potential neuroprotective effects pertinent to Alzheimer's disease (AD) *in vitro*. However, the therapeutic potential of triptolide for AD *in vivo* has not been thoroughly evaluated. In the present study, we investigated the impact of peripherally administered triptolide on AD-related behavior and neuropathology in APP_swe_/PS1_ΔE9_ (APP/PS1) mice, an established model of AD. Our results showed that two-month treatment with triptolide rescued cognitive function in APP/PS1 mice. Immunohistochemical analyses indicated that triptolide treatment led to a significant decrease in amyloid-β (Aβ) deposition and neuroinflammation in treated mice. In contrast to previous findings *in vitro*, biochemical analyses showed that triptolide treatment did not significantly affect the production pathway of Aβ *in vivo*. Intriguingly, further analyses revealed that triptolide treatment upregulated the level of insulin-degrading enzyme, a major Aβ-degrading enzyme in the brain, indicating that triptolide treatment reduced Aβ pathology by enhancing the proteolytic degradation of Aβ. Our findings demonstrate that triptolide treatment ameliorates key behavioral and neuropathological changes found in AD, suggesting that triptolide may serve as a potential therapeutic agent for AD.

## Introduction

Alzheimer's disease (AD) is the most common neurodegenerative disease and a major form of dementia characterized clinically by progressive cognitive impairment [1]. Pathological hallmarks of AD include deposits of aggregated amyloid-β protein (Aβ), neurofibrillary tangles (NFTs), and gliosis in the brain. Mounting evidence indicates an involvement of inflammation in the pathogenesis of AD and accumulating studies have begun to focus on the roles of anti-inflammatory and immune-modulatory agents in AD [2], [3].

Triptolide is the major constituent of the Chinese herb, *Tripterygium wilfordii Hook F* (TWHF), which has been used to treat inflammatory diseases for centuries. Clinical and experimental studies have demonstrated that triptolide has anti-inflammatory, immunosuppressive, and anti-tumor activities [4]. Owing to its small molecular size and lipophilic characteristics, the therapeutic potential of triptolide for diseases of the central nervous system (CNS) has attracted worldwide attention [5].

Recent studies suggested that triptolide influences multiple aspects of AD in various cell models. In microglial cultures, triptolide was found to effectively suppress oligomeric Aβ induced production of pro-inflammatory cytokines, including tissue necrosis factor-α (TNF-α), interleukin-1β (IL-1β), and nitric oxide (NO) species [6]. It also has been shown to inhibit lipopolysaccharide (LPS)-induced cyclooxygenase-2 (COX-2) expression and prostaglandin E2 (PGE2) biosynthesis by suppressing the activity of nuclear factor kappa-B (NF-κB) and c-jun NH_2_-terminal kinase (JNK) [7]. Moreover, triptolide was able to reduce Aβ42 production in cell models of AD by inhibiting the expression of chemokine receptor CXCR2 and presenilin [8]. In addition, triptolide also exhibited neurotrophic activity *in vitro*. Triptolide treatment selectively upregulated the synthesis and release of nerve growth factor (NGF) in primary rat astrocyte cultures [9]. Nie et al. [10] observed that triptolide promoted synaptophysin expression in hippocampal neurons in an AD cellular model. Several *in vivo* studies have also shown that triptolide possesses significant neuroprotective effects. Administration of triptolide significantly delayed the onset of experimental autoimmune encephalomyelitis (EAE) and suppressed the disease severity through induction of HSP70 and stabilization of the NF-κB/IκB transcriptional complex in an induced EAE mouse model [11]. Gao et al. [12] reported that triptolide prevented dopaminergic neuron loss through the inhibition of microglial activation in a hemi-parkinsonian rat model. However, the therapeutic potential of triptolide for AD has not been thoroughly evaluated *in vivo*.

In the present study, we investigated the effects of triptolide on AD-like behavior and neuropathology in APP_swe_/PS1_ΔE9_ (APP/PS1) mice, an established model of AD [13]. Our results demonstrate that triptolide treatment rescued spatial memory deficits, reduced Aβ deposition, and attenuated neuroinflammation in APP/PS1 mice.

## Materials and Methods

### Animals and treatments

Breeders of APP/PS1 double transgenic (Tg) mice (B6C3-Tg (APPswe, PSEN1dE9) 85Dbo/J; stock number 004462) were purchased from the Jackson Laboratory (Bar Harbor, ME) and bred with B6C3F1 mice to establish a colony of APP/PS1 mice and non-Tg littermates. In this study, 5 month old female APP/PS1 mice were randomly assigned into two groups: the treated group (Triptolide) and the untreated group (Control). Female APP/PS1 mice were used because they develop AD-related neuropathology at an earlier age and more extensively than male APP/PS1 mice [14]. The treated group was first given triptolide via diet administration (added as a diet admixture so that approximately 0.4 mg triptolide/kg body weight was consumed daily). After 10 days of the treatment, the treated mice showed significant loss of body weight and three mice were found dead. The treatment was discontinued to allow the washout of drug effect. After 18 days of drug withdrawal, the body weight of all mice was restored to the pretreatment level. Subsequently, the dose of triptolide was reduced to 0.2 mg/kg body weight via intraperitoneal injection, the safe treatment regimen used in a previous study [15]. The triptolide was dissolved in 5% DMSO in saline and the treated mice were injected daily at 0.2 mg/kg for 2 months and subjected to behavioral assessments. The control group received an equal volume of 5% DMSO in saline. Untreated non-Tg littermates were included as wild type controls. This study was carried out in strict accordance with the recommendations in the Guide for the Care and Use of Laboratory Animals of the National Institutes of Health. All animal procedures were prospectively reviewed and approved by the Institutional Animal Care and Use Committee of the University of Minnesota (Protocol number: 1308–30854A). All efforts were made to minimize suffering.

### Behavioral assessment

Three AD-related behavioral functions (spatial learning and memory, exploration of environmental stimuli, and anxiety) were assessed. The testing schedule included the open field test for locomotor activity (days 1–3), the elevated plus-maze test for anxiety levels (days 4 and 5), and spatial learning in the Morris water maze (days 6–11). All equipment and software were purchased from SD Instruments (San Diego, CA). All testing procedures have been described in detail previously [16], [17].

### Blood collection and brain tissue preparation

The mice were deeply anesthetized with an intraperitoneal injection of ketamine (100 mg/kg) and xylazine (10 mg/kg). After confirming the complete depression of palpebral and pedal reflexes in anesthetized mice, blood was collected by cardiac puncture with heparin as an anticoagulant. Following perfusion via the heart with ice-cold PBS, brains were cut sagittally into left and right hemispheres. The left hemisphere was fixed in 4% paraformaldehyde for histological analysis. The right hemisphere (devoid of cerebellum and brain stem) was snap frozen in liquid nitrogen and stored at −80°C for biochemical analysis.

### Brain Aβ ELISA and immunoblot analysis

Brain homogenates were prepared as we described previously [16], [17]. Commercial ELISA kits (Invitrogen) were used to measure Aβ40 and Aβ42 levels in carbonate soluble and insoluble (guanidine soluble) fractions according to the manufacturer's protocol. For immunoblot analysis, aliquots of brain homogenate were separated by SDS-PAGE and blotted to nitrocellulose or polyvinylidene difluoride (PVDF) membranes. The membranes were incubated with specific primary antibodies against the carboxyl terminus of APP (CT695; Invitrogen), the soluble amino-terminal fragments of APP (sAPPα and sAPPβ) (2B3 (11088B) and 6A1 (10321B), Clontech), mouse apolipoprotein E (apoE) (sc-6384, Santa Cruz Biotechnology), insulin-degrading enzyme (IDE) (PC730, EMD Biosciences/Millipore), neprilysin (sc-9149, Santa Cruz Biotechnology), ionized calcium-binding adaptor molecule 1 (IBA1) (016-20001, Wako), and nitric oxide synthase 2 (NOS2) (sc-7271, Santa Cruz Biotechnology) followed by HRP-conjugated secondary antibodies. Signal was detected by the ECL plus Western Blotting System (GE Healthcare) and quantified by ImageJ software. For a loading control when appropriate, the blots were stripped and reprobed with mouse anti-actin antibody (MAB1501R, Millipore) or mouse anti-tubulin antibody (T5168; Sigma).

### Immunohistochemical analysis and quantification of Aβ deposition and activated microglia

Protocols for immunohistochemical analysis have been described previously [16], [17]. Briefly, fixed brain tissues were sectioned at 50 µm using a vibratome (Leica Microsystems Inc). Tissue sections were stored at 4°C in PBS with 0.01% sodium azide and subjected to free-floating immunostaining using the ABC kit (Vector, Burlingame, CA) to detect Aβ and activated microglia. The primary antibody 6E10 (Covance) was used for assessing Aβ deposition and IBA-1 antibody (Wako) for assessing activated microglia. Immunoreactivity of Aβ and IBA1 in the cortex and hippocampus of the mouse brain was quantified using a histomorphometry system (Image-Pro Plus, MediaCybernetics, Rockville, MD).

### Statistical analysis

Data are expressed as means ± standard error (SE). Comparison of different groups was performed by Student's t-test and repeated measures analysis of variance. P<0.05 was considered statistically significant.

## Results

### Triptolide rescued learning and memory deficits in APP/PS1 Mice

**Figure 1:**
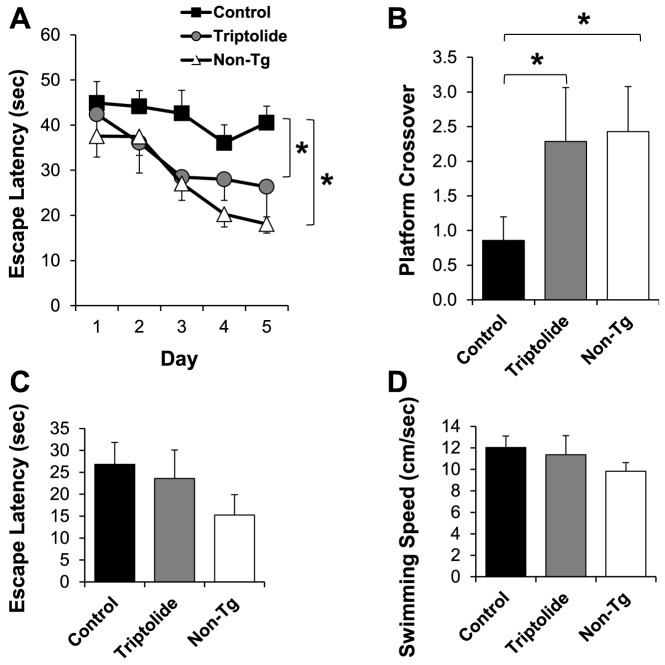
Triptolide treatment rescues spatial learning and memory deficits in APP/PS1 mice. ***A***, Escape latency during the acquisition phase of the Morris water maze test. Untreated control APP/PS1 mice exhibited learning deficits in locating the submerged escape platform, whereas triptolide-treated mice performed similarly to non-Tg mice. ***B***, The number of crossings over the previously hidden platform area in the probe trial. Triptolide-treated group crossed the platform area significantly more often than control APP/PS1 group. ***C*** and ***D***, Escape latency and swimming speed during the visible platform phase of the Morris water maze test. No significant differences were observed among different groups. For clarity, error bars are shown in only one direction. N = 7 mice/group; Age = 8 months; *, *P*<0.05.

To assess the effect of triptolide treatment on spatial learning and memory ability of the mice, the Morris water maze test was conducted. The results show that, in the acquisition phase (**Fig 1A**), non-Tg mice readily learned the location of the hidden platform in the course of the 5-day trial, whereas APP/PS1 control mice portrayed an inability to find the platform. Remarkably, triptolide-treated APP/PS1 mice exhibited a substantial improvement in learning and memory function as proven by the decrease in escape latency across trials, similar to normal non-Tg mice. The cognitive improvement in triptolide-treated APP/PS1 mice was also evidenced by the probe trial for memory retention. As the non-Tg mice, triptolide-treated APP/PS1 mice crossed over the previous platform location significantly more often than untreated control APP/PS1 mice (**Fig 1B**). In the visible platform test of the Morris water maze, triptolide treated and untreated APP/PS1 mice performed similarly (**Fig. 1C, D**), indicating that there were no changes of visual acuities and swimming strategies associated with the triptolide treatment.

### Triptolide treatment restored habituation of APP/PS1 mice in an open field but had no significant effect on anxiety levels

**Figure 2:**
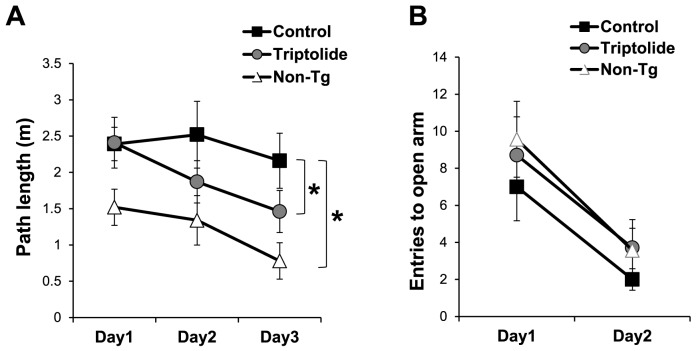
Triptolide treatment restores intersessional habituation in APP/PS1 mice. ***A*,**Untreated control APP/PS1 mice were more active than mice in any other groups and did not show intersessional habituation. Triptolide-treated APP/PS1 mice, in contrast, performed similarly as non-Tg mice and displayed intersessional habituation shown by decreasing activity in the latter 2 days of testing. ***B*** **,** Triptolide treatment had no effect on anxiety levels in the elevated plus maze tests. N = 7 mice/group; Age = 8 months; *, *P*<0.05.

Prior to the Morris water maze test, the mice were assessed for motor activity in an open field and anxiety in an elevated plus-maze. In the open field test, there were no differences in activity levels among different groups of mice on day 1 (**Fig 2A**). Untreated control APP/PS1 mice, however, were more active than non-Tg mice during the next 2 days (p<0.05), whereas triptolide-treated APP/PS1 mice showed significant day effects for path length (p<0.05), decreasing activity in the latter 2 days as a result of habituation (**Fig 2A**), similar to non-Tg mice. Thus, triptolide treatment restored habituation of APP/PS1 mice in the open field. In the elevated plus maze tests, no differences were observed in anxiety levels among different groups (**Fig 2B**).

### Triptolide treatment reduced steady state levels and deposition of Aβ in brains of APP/PS1 mice

**Figure 3:**
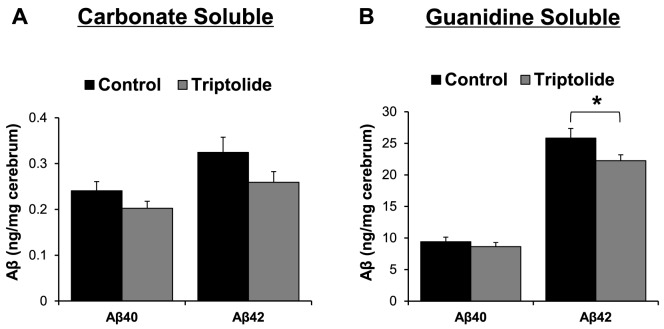
Triptolide treatment reduces cerebral Aβ levels in APP/PS1 mice. Carbonate-soluble (***A***) and carbonate-insoluble (guanidine-soluble) (***B***) Aβ_40_ and Aβ_42_ levels in cerebral homogenates were measured by ELISA. ***A***, There was a trend for a reduced level of carbonate-soluble Aβ in triptolide-treated mice, but the difference did not reach statistical significance. ***B***, Significant reductions in the levels of guanidine-soluble Aβ_42_ were observed in triptolide-treated mice. N = 7 mice/group; Age = 8.6 months; *, *P*<0.05.

**Figure 4:**
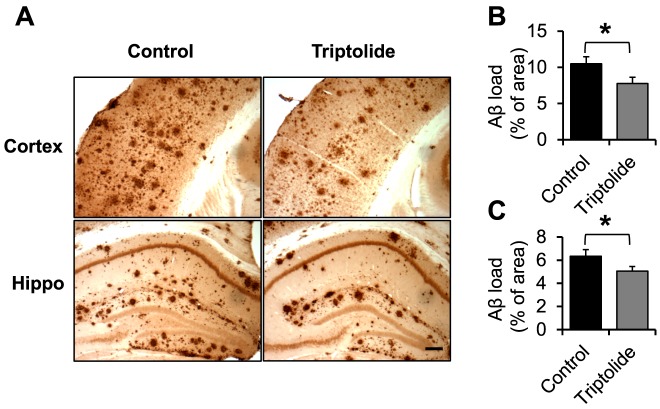
Triptolide treatment significantly decreases cerebral Aβ plaque load in APP/PS1 mice. ***A***, Representative brain sections of cortical and hippocampal areas from different groups immunostained with anti-Aβ antibody (6E10). ***B, C***, Quantification of the percent amyloid load in the cortical (*B*) and hippocampal (*C*) areas, showing a significant reduction in the triptolide-treated group when compared to the control group. N = 7 mice/group; Age  = 8.6 months; *, *P*<0.05. Scale bars  = 100 µm.

To define the impact of triptolide treatment on cerebral β-amyloidogenesis in APP/PS1 mice, the levels of Aβ in the carbonate-soluble and -insoluble (guanidine-soluble) fractions in brain lysates were measured by Aβ40 and Aβ42 specific ELISA. In the carbonate-soluble fraction, there was a trend for reduction in Aβ levels in triptolide-treated mice compared to control APP/PS1 mice (**Fig 3A**). In the guanidine-soluble fraction, the levels of Aβ42 were significantly reduced in triptolide-treated mice when compared with APP/PS1 mice (**Fig 3B**). Consistent with these findings, immunohistochemical analyses showed that Aβ deposition was significantly lower in triptolide-treated mice than in control APP/PS1 mice in both cortical and hippocampal area (**Fig 4A, B**).

### Triptolide did not affect APP processing but upregulated the degradation pathway of Aβ in APP/PS1 mice

**Figure 5:**
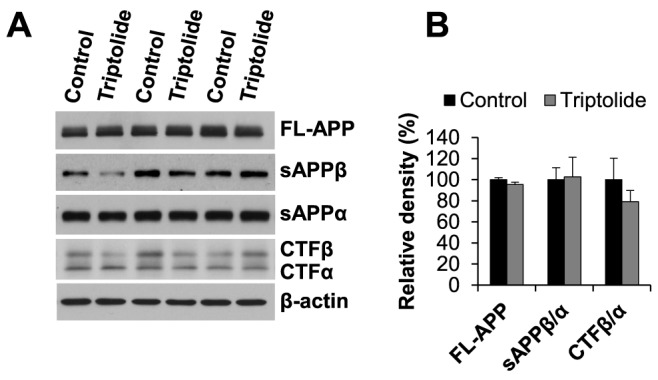
Triptolide treatment does not affect APP processing. ***A***, Immunoblot analysis of full-length APP (FL-APP), carboxyl-terminal fragments (CTFα and CTFβ), and amino-terminal fragment of APP (sAPPα and sAPPβ). ***B***, Densitometric analysis of immunoblots (normalized by the amount of β-actin) with the levels in the control group set as 100%. There were no differences in the amount of FL-APP, the ratio of β-CTF to α-CTF, and the ratio of sAPPβ to sAPPα between triptolide treated and control APP/PS1 mice. N = 7 mice/group; Age  = 8.6 months.

To elucidate the mechanisms by which triptolide reduced the level and deposition of Aβ, the impact of triptolide treatment on Aβ production and clearance was studied. It was reported recently that triptolide could reduce Aβ42 production in cell models of AD [8]. To investigate whether triptolide could affect APP processing in APP/PS1 mice, the steady-state levels of full-length APP (FL-APP), carboxyl-terminal fragments (CTF), and soluble amino-terminal fragments of APP (sAPPα and sAPPβ) produced by α- and β-secretase cleavages were measured by immunoblot analyses. The results showed that there were no significant differences in the amount of FL-APP, the ratio of CTFβ to CTFα, and the ratio of sAPPβ to sAPPα between triptolide-treated and control APP/PS1 mice (**Fig 5A, B**). Interestingly, there was a trend of decrease in the ratio of CTFβ to CTFα in triptolide-treated mice but it did not reach statistical significance. The findings suggest that triptolide treatment does not significantly affect Aβ production.

**Figure 6:**
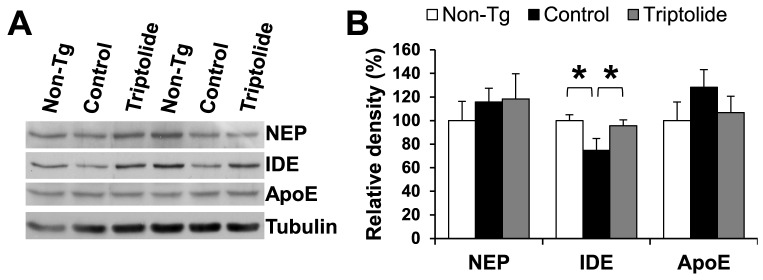
Effect of triptolide treatment on proteins involved in clearance/degradation of Aβ in APP/PS1 mice. ***A***, Immunoblot analysis of ApoE, NEP, and IDE levels in the cerebral homogenates. ***B***, Densitometric analysis of immunoblots (normalized by the amount of tubulin), with the levels in the non-Tg group set as 100%. The level of IDE was significantly decreased in control APP/PS1 mice but restored to the normal level in triptolide-treated APP/PS1 mice. N = 7 mice/group; Age  = 8.6 months; *, *P*<0.05.

Next, the factors related to Aβ clearance and degradation were examined. Insulin-degrading enzyme (IDE) and neprilysin (NEP) are two major enzymes involved in the degradation of Aβ in the brain [18]. Notably, reduced IDE and NEP levels have been found in the brains of AD patients and in mouse models of AD [19]. To determine if triptolide treatment affected the level of IDE and NEP, brain tissue lysates of treated and control APP/PS1 mice were subjected to immunoblot analyses. Samples from non-Tg littermates were also included for comparison. The results showed that the level of IDE was significantly reduced in control APP/PS1 mice compared to non-Tg littermates but was restored to the normal level in triptolide-treated APP/PS1 mice (**Fig. 6A, B**). The level of NEP was unchanged among different groups. The level of ApoE, one of major proteins involved in the clearance of Aβ in the brain, was also measured, and no significant change was found among different groups of mice. These findings suggest that triptolide treatment enhances Aβ degradation via the upregulation of IDE.

### Triptolide attenuated neuroinflammation in APP/PS1mice

**Figure 7:**
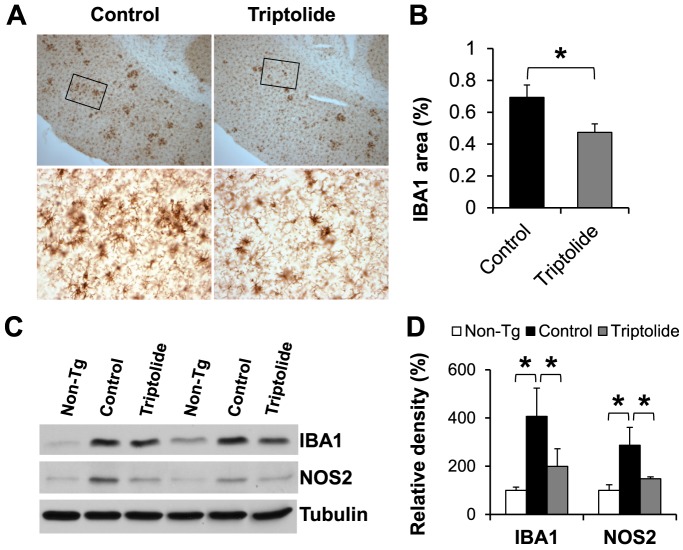
Triptolide treatment attenuates neuroinflammation in APP/PS1 mice. ***A***, Representative photomicrographs of activated microglia stained with IBA1 antibody. ***B***, Quantification of percent IBA1 immunoreactivity in the cortex of triptolide-treated and control mice. ***C***, Immunoblot analysis of IBA1 and NOS2 levels in the cerebral homogenates. ***D***, Densitometric analysis of IBA1 and NOS2 immunoblots (normalized by the amount of tubulin), with the levels in the non-Tg group set as 100%. The levels of IBA1 and NOS2 were elevated in control APP/PS1 mice but significantly attenuated in triptolide-treated APP/PS1 mice. N = 7 mice/group; Age  = 8.6 months; *, *P*<0.05.

Aberrant inflammatory responses, such as activation of microglia, are common pathological features in the brains of AD patients. To assess the impact of triptolide on neuroinflammation, the extent of microglial activation was determined by immunohistochemical analysis of IBA1, a marker of microgliosis, in the brain of triptolide-treated and control APP/PS1 mice. Quantitative analysis indicated that microgliosis was significantly decreased in the cortex of triptolide-treated mice compared to control APP/PS1 mice (**Fig 7A, B**). Consistently, immunoblot analyses of IBA1 in brain homogenates showed similar results as the immunohistochemical analyses (**Fig 7C, D**). The level of IBA1 in control APP/PS1 mice was elevated compared to non-Tg littermates but was significantly reduced in triptolide-treated APP/PS1 mice. These findings demonstrate that triptolide treatment reduces microglial activation.

Increased levels of the inducible nitric oxide synthase (NOS2) have been implicated in the response to the inflammatory stimuli in AD, potentially aggravating disease progression [20]. Consistently, the level of NOS2 was significantly elevated in the brain of control APP/PS1 mice compared to non-Tg littermates (**Fig 7C, D**). Triptolide was found to effectively suppress the NOS2 expression and LPS-induced NO accumulation in microglial cultures [21]. In agreement with these *in vitro* findings, the level of NOS2 in the brain of triptolide-treated APP/PS1 mice was significantly decreased compared to control APP/PS1 mice (**Fig 7C, D**), suggesting the downregulation of NOS2 as a potential mechanism for anti-inflammatory effects of triptolide.

## Discussion

Cognitive impairment is the chief clinical manifestation of AD and unarguably the central target of any drug therapy. Triptolide has been shown to display neuroprotective effects in different animal models and to affect multiple aspects of AD in various *in vitro* cell models. Chronic effects of triptolide on memory impairment and Aβ-related pathology *in vivo* had not been reported in peer-reviewed literature. The present study demonstrates that peripheral administration of triptolide rescued spatial learning and memory deficits observed in APP/PS1 mice. The cognitive dysfunction associated with these mice is a result of an accelerated accumulation of Aβ and loss of functional synapses [22]–[24]. It is believed that such loss is partly caused by activated glial cells that target Aβ deposits for degradation but in the process can damage neurons in and around the deposits. In the present study, treatment of APP/PS1 mice at 5 months of age with triptolide led to a significant decrease in Aβ deposition and neuroinflammation, which concurrently prevented learning and memory deficits in these mice.

How triptolide exerts beneficial effects is not fully understood. Previous studies *in vitro* have showed that triptolide modulates multiple aspects of AD-related processes. A recent study reports that triptolide inhibits the expression of β- and γ-secretases, and reduces the production of both Aβ40 and Aβ42 with high potency in cell model of AD [8]. In the present study, there was a trend for reduction in the level of Aβ40 and Aβ42 in the carbonate soluble fraction and a significant decrease of Aβ42 in the insoluble fraction. Interestingly, the steady state levels of FL-APP, CTFα, CTFβ, sAPPα, and sAPPβ, which are regulated by secretases, were not changed significantly in triptolide-treated APP/PS1 mice, indicating that triptolide treatment does not influence amyloidogenic processing of APP in APP/PS1 mice. Of note, there was a trend of decrease in the ratio of of CTFβ to CTFα in triptolide-treated mice, which could be caused by an increase in degradation of CTFβ or in the formation of CTFα. It has been reported that CTFβ is subjected to degradation by proteosomal, lysosomal, and autophagosomal pathways independent of γ-secretase [25]–[27]. In addition, CTFβ can be converted to CTFα by α-secretase [28], [29]. Thus, it is possible that triptolide treatment enhances the CTFβ degradation pathway and/or the conversion of CFTβ to CTFα, resulting in a trend of decrease in the ratio of CTFβ to CTFα. The significance of such potential effects by triptolide warrants further investigation.

Intriguingly, triptolide treatment resulted in a significant increase in the level of IDE, a key enzyme for Aβ degradation in the brain. How triptolide upregulates IDE is not clear. It has been shown that NOS2-mediated chronic NO formation decreases the activity of IDE in mice [30]. Triptolide has been shown to effectively suppress NOS2 expression and reduce NO accumulation in LPS-induced microglial cultures [21]. Consistent with these findings, the present study showed that triptolide treatment substantially decreased the level of NOS2 in the brain of APP/PS1 mice, which might be partly responsible for the upregulation of IDE in these mice.

Numerous studies have strongly suggested that chronic neuroinflammation plays a critical role in the pathogenesis and cognitive dysfunction of Alzheimer's disease [2]. The inflammatory response in AD is a “double-edged sword”. Microglia are recruited to sites of Aβ deposition as part of the attempt of the brain to clear these neurotoxic peptides, and early microglial activation in AD delays disease progression. However, during aging, microglia become dysfunctional and show a significant reduction in expression of their Aβ-binding receptors and Aβ-degrading enzymes, but maintain their ability to produce proinflammatory cytokines and neurotoxins, hence promoting neurodegeneration [31], [32]. While triptolide-mediated reduction of Aβ deposition would lead to less inflammation in the brain, triptolide has also been shown to possess direct anti-inflammatory properties. In microglial culture, triptolide inhibits the production of inflammatory cytokines [6], and suppresses the activity of NF-κB and related inflammatory pathways [7]. In neuronal cells, triptolide downregulates the expression of a chemokine receptor CXCR2 and inhibits its downstream effects [8]. Recent studies have identified the molecular target of triptolide to be XPB, a subunit of the transcription factor TFIIH [33]. Triptolide inhibits the DNA-dependent ATPase activity of TFIIH, leading to the degradation of RNA polymerase II [33], [34]. This action explains the majority of biological effects of triptolide, including the inhibitory effect of triptolide on the activity of a number of transcription factors such as NF-κB [33]. As the expression of NOS2 is controlled by NF-κB [35], the decrease in NOS2 in triptolide-treated APP/PS1 mice most likely resulted from the inhibition of NF-κB by triptolide via its action on XPB/TFIIH, counteracting the increase of neuroinflammation in these AD mice.

In addition to reduction of Aβ deposition and attenuation of neuroinflammation, triptolide might exert other neuroprotective effects that contributed to the preservation of learning and memory function in APP/PS1 mice. Some studies have shown that triptolide inhibits glutamate and Ca^2+^-induced excitotoxicity in neuronal cells. The underlying mechanisms may include the inhibition of reactive oxygen species (ROS) formation and the decrease of mitochondrial membrane potential [36], [37]. In addition, triptolide has been shown to display neurotropic effects *in vitro*. Triptolide treatment selectively upregulates the synthesis and release of NGF in primary rat astrocyte cultures [9], and increases the level of synaptophysin in hippocampal neurons [10].

Although triptolide treatment results in multiple beneficial effects in APP/PS1 mice, it is important to note that at a high dose, triptolide had severe adverse effects in these mice, as observed initially in the present study. Also, triptolide is poorly soluble in water, which limits its potential clinical use. To this end, a water-soluble prodrug of triptolide, Minnelide, has been developed, which has been shown to be as effective as triptolide against pancreatic cancer in preclinical animal models [15]. Minnelide is currently being tested in a clinical trial for patients with advanced gastrointestinal tumors (ClinicalTrials.gov; NCT01927965). Whether Minnelide works as triptolide in AD mice awaits further investigation.

In conclusion, the current study provides preclinical evidence that chronic triptolide treatment reduces Aβ deposition, attenuates neuroinflammation, and prevents cognitive deficits in a mouse model of AD. These results suggest that triptolide may serve as a potential therapeutic agent for AD. However, it remains to be determined whether this compound displays the pharmacokinetic characteristics and toxicity profile required for a drug candidate.
